# Supplementary material for: Identification and expression analysis of miRNAs and elucidation of their role in salt tolerance in rice varieties susceptible and tolerant to salinity
Source: PLoS One. 2020 Apr 15;15(4):e0230958. doi: 10.1371/journal.pone.0230958 (PMC7159242; doi:10.1371/journal.pone.0230958)
Supplement: S1 File — (DOCX) [file pone.0230958.s001.docx]

**S1 File**


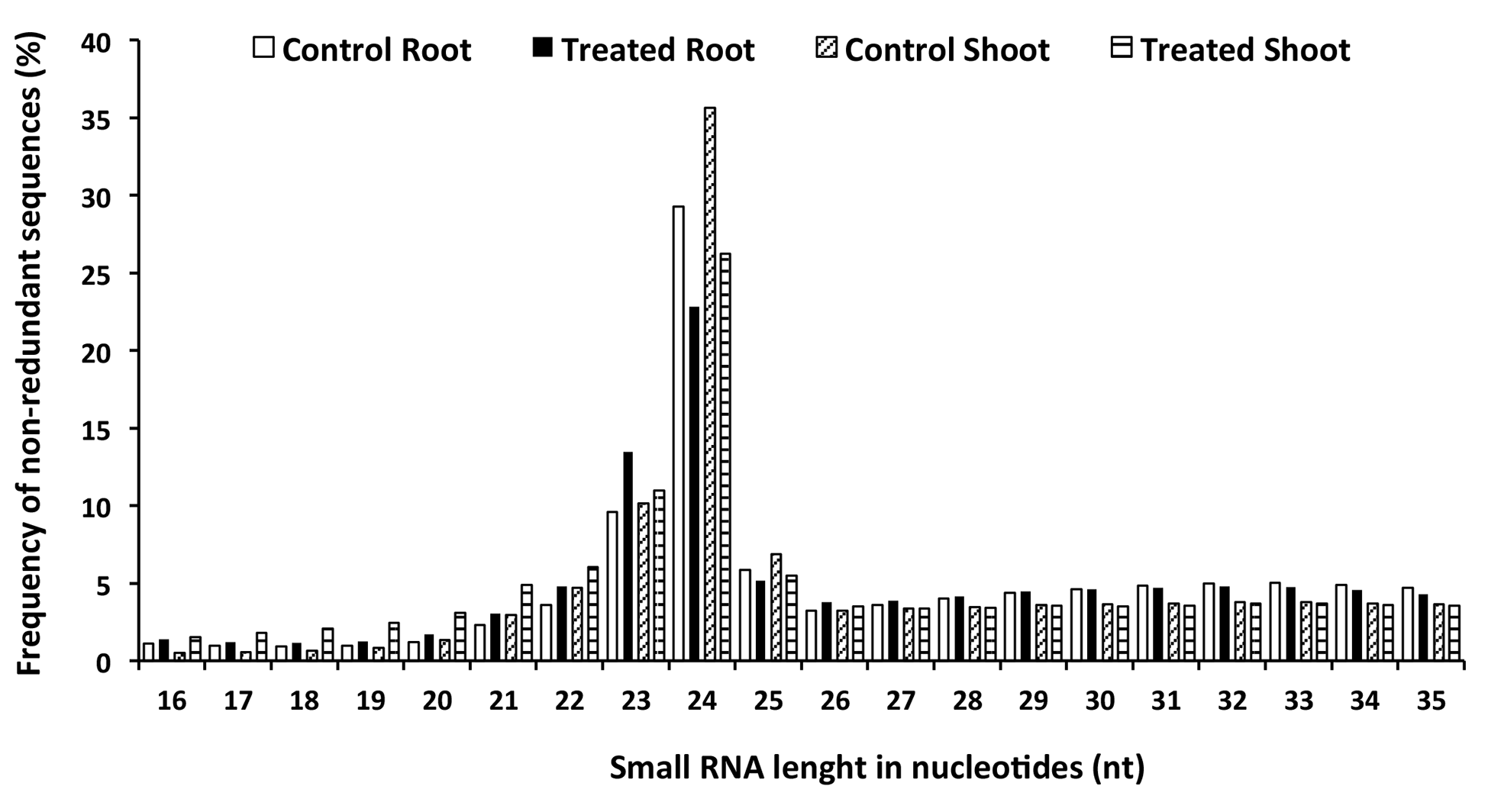


**Frequency of non-redundant sequences (%) of sRNA ranging from 16 nucleotides (nt) to 29-nt in control and 1.5 % NaCl treated root and shoot *O. sativa* CV. Pokkali seedlings.** Treatment applied on 9^th^ day of germination. The frequency was obtained as percentage of fraction of putative miRNA reads of a particular length (nt) to the total number of putative miRNA reads.

The non-redundant reads of length between 16-nt and 35-nt were 1143060, 1664461, 1219751 and 1035994, respectively for control root (CR), control shoot (CS), treated root (TR) and treated shoot (TS). Most of the sRNA non-redundant sequences from all the libraries were 23-nt to 24-nt long. The representation from 20-nt to 23-nt, to which miRNAs/siRNAs belong, was ~20 %. These nearly constituted more than 45 % of the total redundant sequences. Salt treatment revealed increase in abundance of all these reads in both root and leaves.
