# Supplementary material for: Identification and expression analysis of miRNAs and elucidation of their role in salt tolerance in rice varieties susceptible and tolerant to salinity
Source: PLoS One. 2020 Apr 15;15(4):e0230958. doi: 10.1371/journal.pone.0230958 (PMC7159242; doi:10.1371/journal.pone.0230958)
Supplement: S2 File — (DOCX) [file pone.0230958.s002.docx]

**S2 File**


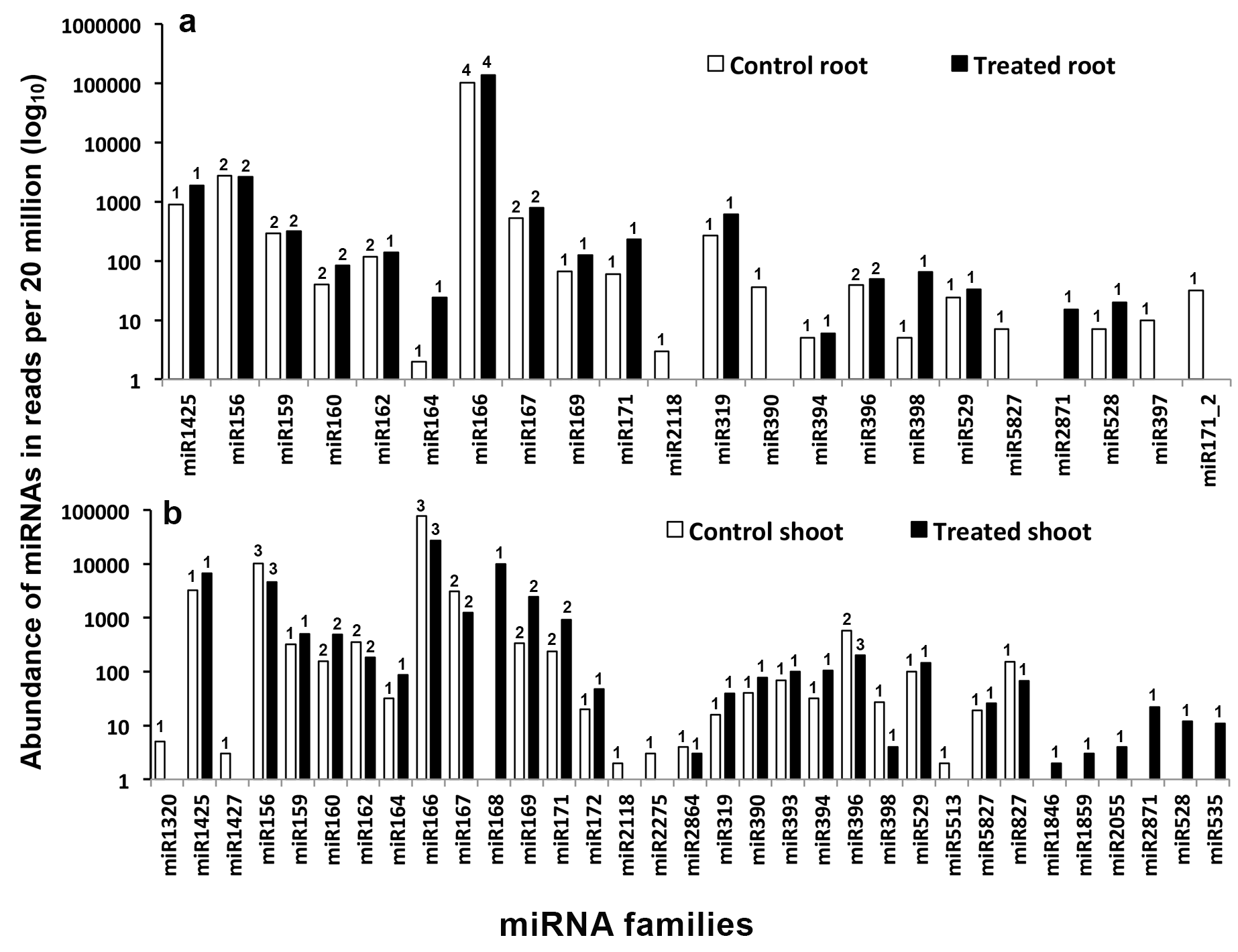


**Abundance of the conserved miRNAs normalized to reads per 20 million in the individual miRNA families in root (a) and shoot (b) of control and 1.5 % NaCl treated seedlings of *O. sativa* cv. Pokkali.** Treatment applied on 9^th^ day of germination. The numerical values at the top of each bar represent the number of individual conserved miRNAs represented by these families in root and shoot tissues out of the total identified.

Both root and shoot showed great difference in composition of the novel miRNAs under control and salt treated conditions. The conserved miRNAs identified belonged to 35 families. The maximum representation of these miRNAs to a family was four, represented by miR166 in root (**a**). The miRNA families miR156, miR166 and miR396 represented three miRNAs each (**b**), while the miRNA families miR160, miR162, miR167, miR171 and miR159 represented two miRNAs each (**a, b**). Together these families represented 46 % of the conserved miRNAs. The other miRNA families represented only one miRNA each. There were miRNA families representing miRNAs specific only to shoot, like miR1320, miR168, miR172, miR2275, miR2864, miR393, miR5827, miR1846, miR1859, miR2055, miR2864 and miR2871. In contrast only two miRNA families, including miR397 miR171_2, represented miRNA only from root. In addition to the tissue specificity, the presence of several miRNAs was also noted to be influenced by salt. Several miRNAs families like miR1846, miR1859, miR2055, miR2871, miR528, miR535 and miR168 represented miRNAs induced in shoot only in response to salt application (**3b**). Similarly, there were several miRNA families, like miR1320, miR1427, miR2118, miR2275, miR397, miR171_2 and miR5513 represented miRNAs expressed only in control condition; salinity treatment silenced their expression. In addition, at least one miRNA family, miR396 showed expression of an additional miRNA in shoot in response to salt exposure. On the other hand the miRNA family miR162 showed silencing of expression of one of its members in root in response to salt exposure.
