# Supplementary material for: Identification and expression analysis of miRNAs and elucidation of their role in salt tolerance in rice varieties susceptible and tolerant to salinity
Source: PLoS One. 2020 Apr 15;15(4):e0230958. doi: 10.1371/journal.pone.0230958 (PMC7159242; doi:10.1371/journal.pone.0230958)
Supplement: S4 File — (PPTX) [file pone.0230958.s004.pptx]

## Slide 1
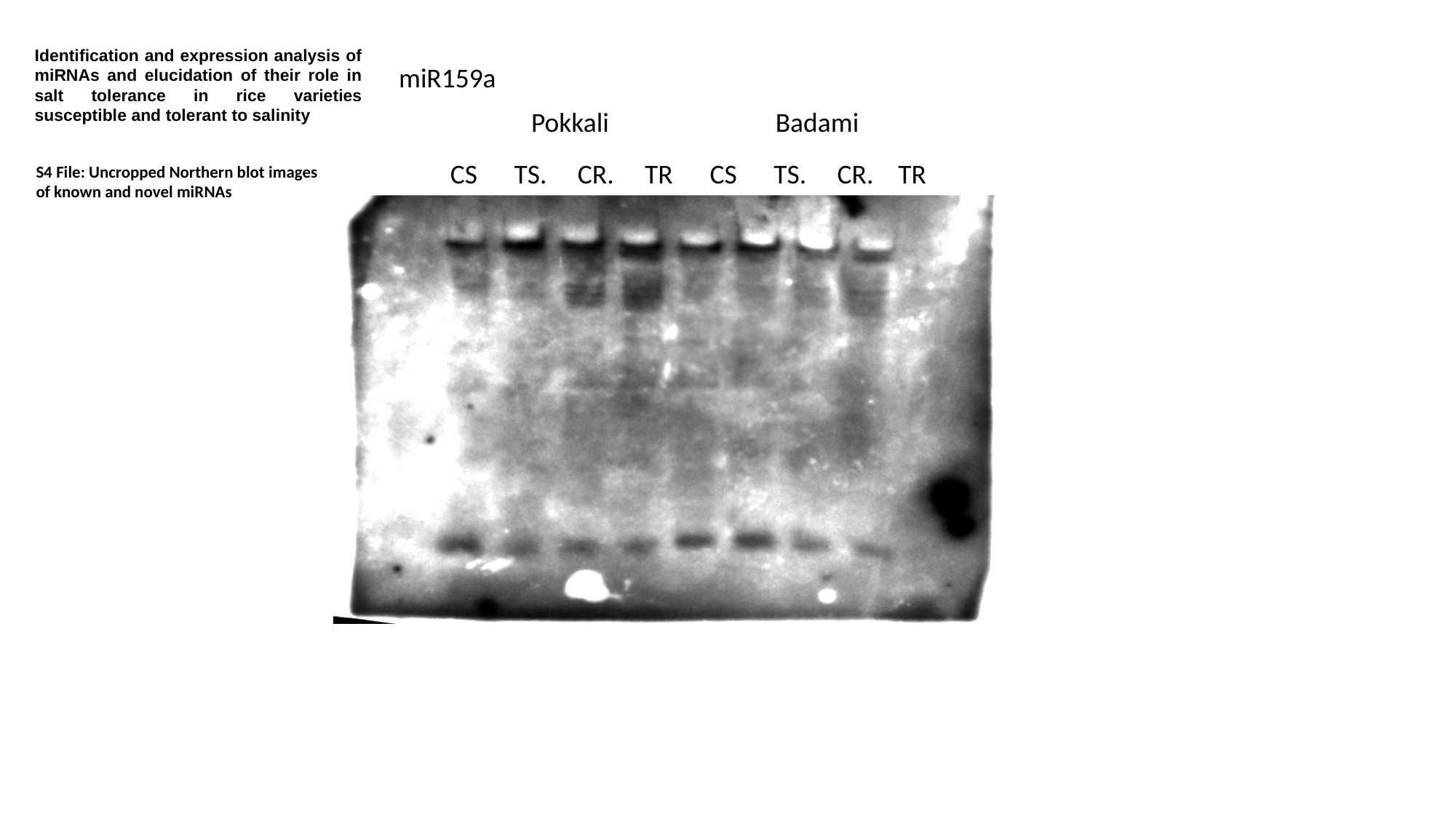

Identification and expression analysis of miRNAs and elucidation of their role in salt tolerance in rice varieties susceptible and tolerant to salinity
miR159a
Pokkali Badami
CS TS. CR. TR CS TS. CR. TR
S4 File: Uncropped Northern blot images
of known and novel miRNAs

## Slide 2
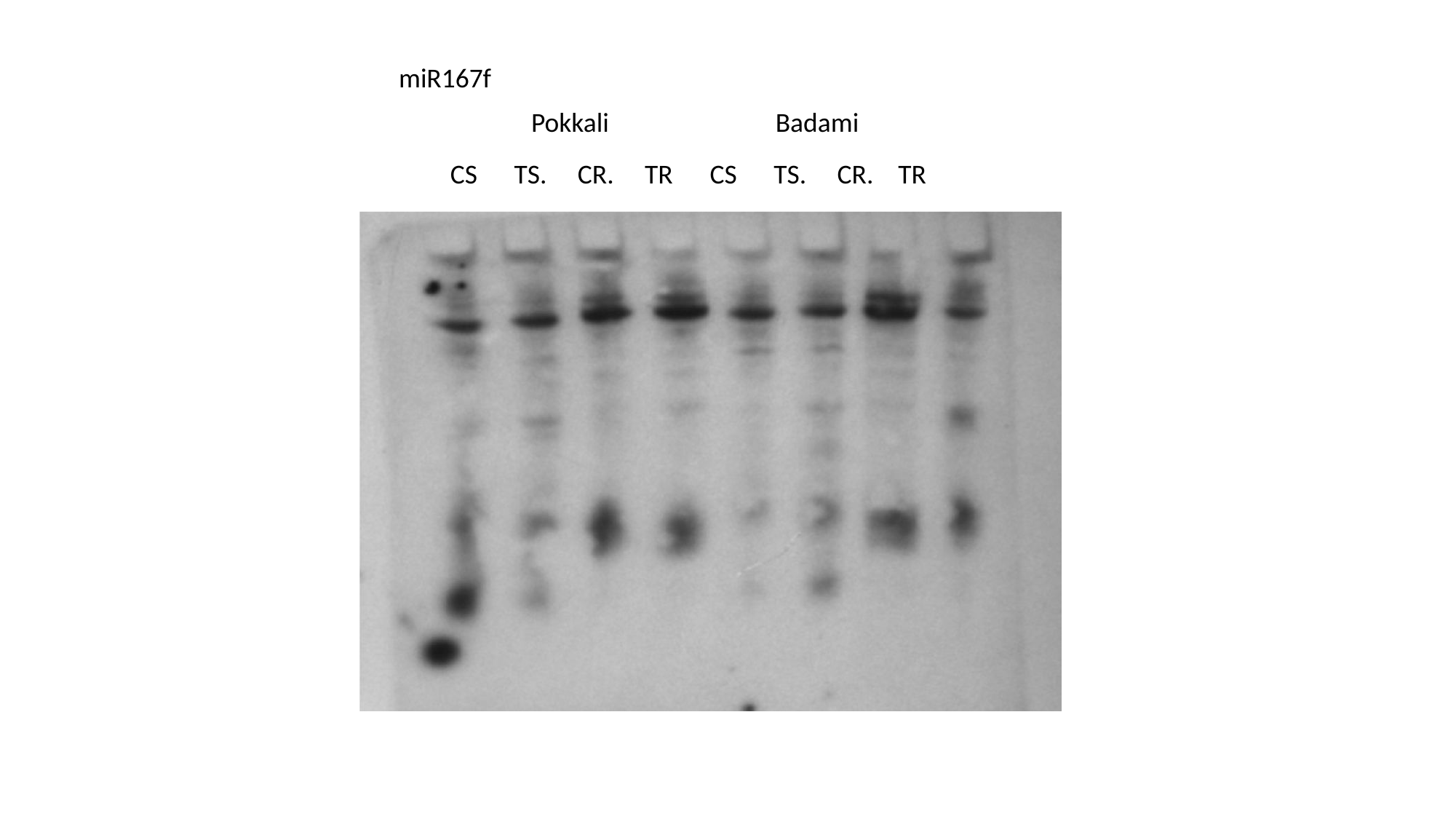

miR167f
Pokkali Badami
CS TS. CR. TR CS TS. CR. TR

## Slide 3
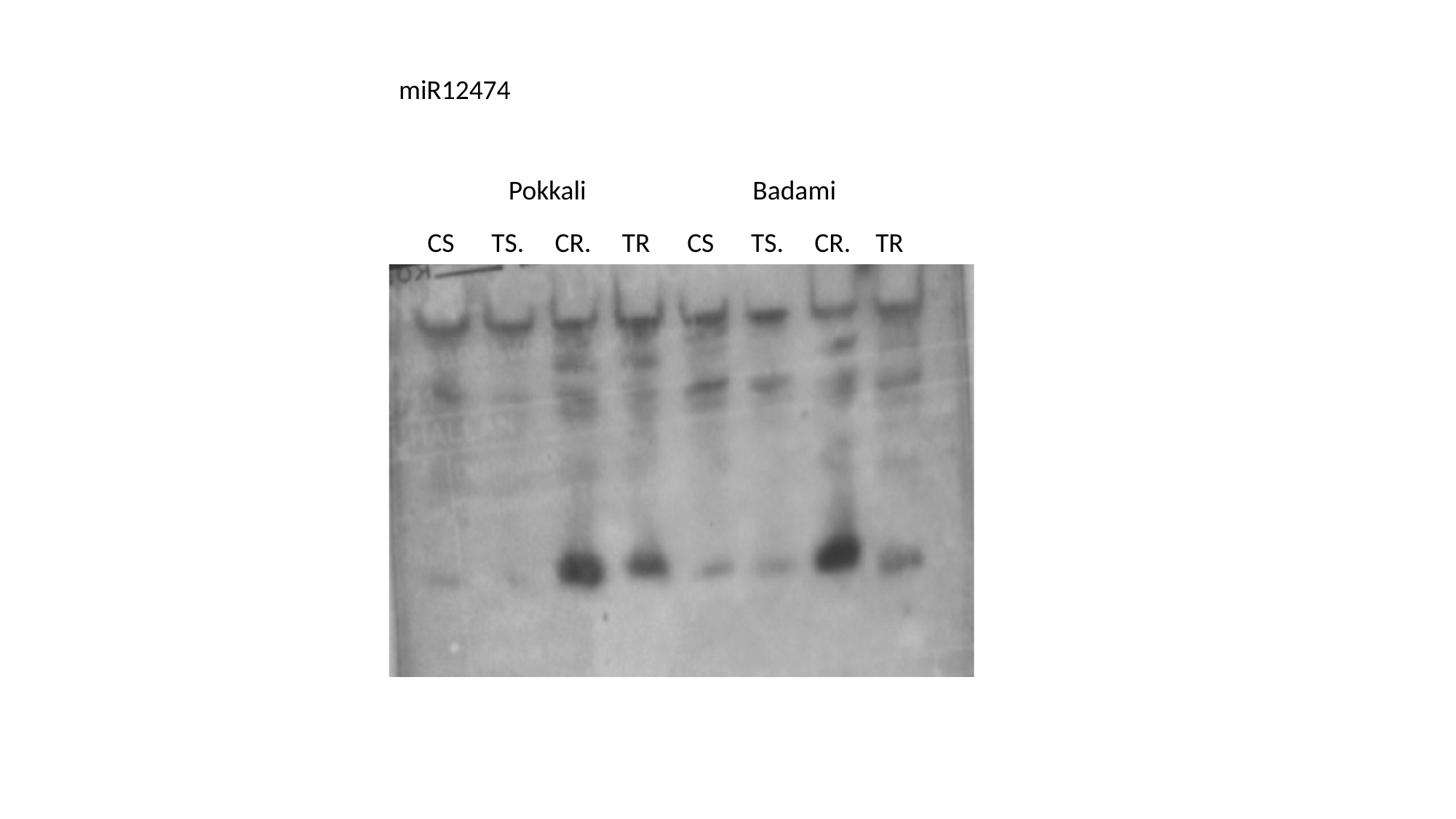

miR12474
Pokkali Badami
CS TS. CR. TR CS TS. CR. TR

## Slide 4
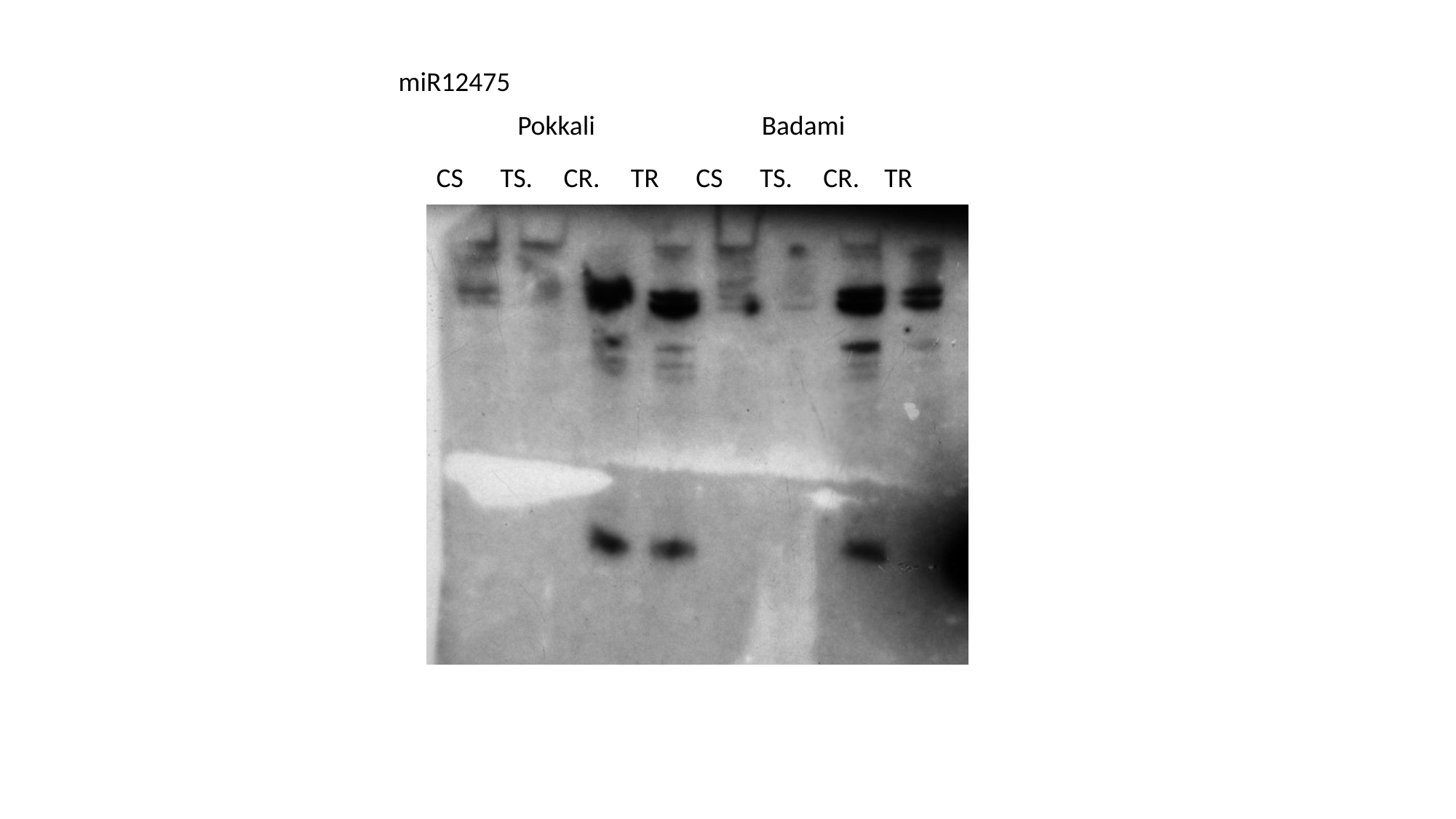

miR12475
Pokkali Badami
CS TS. CR. TR CS TS. CR. TR

## Slide 5
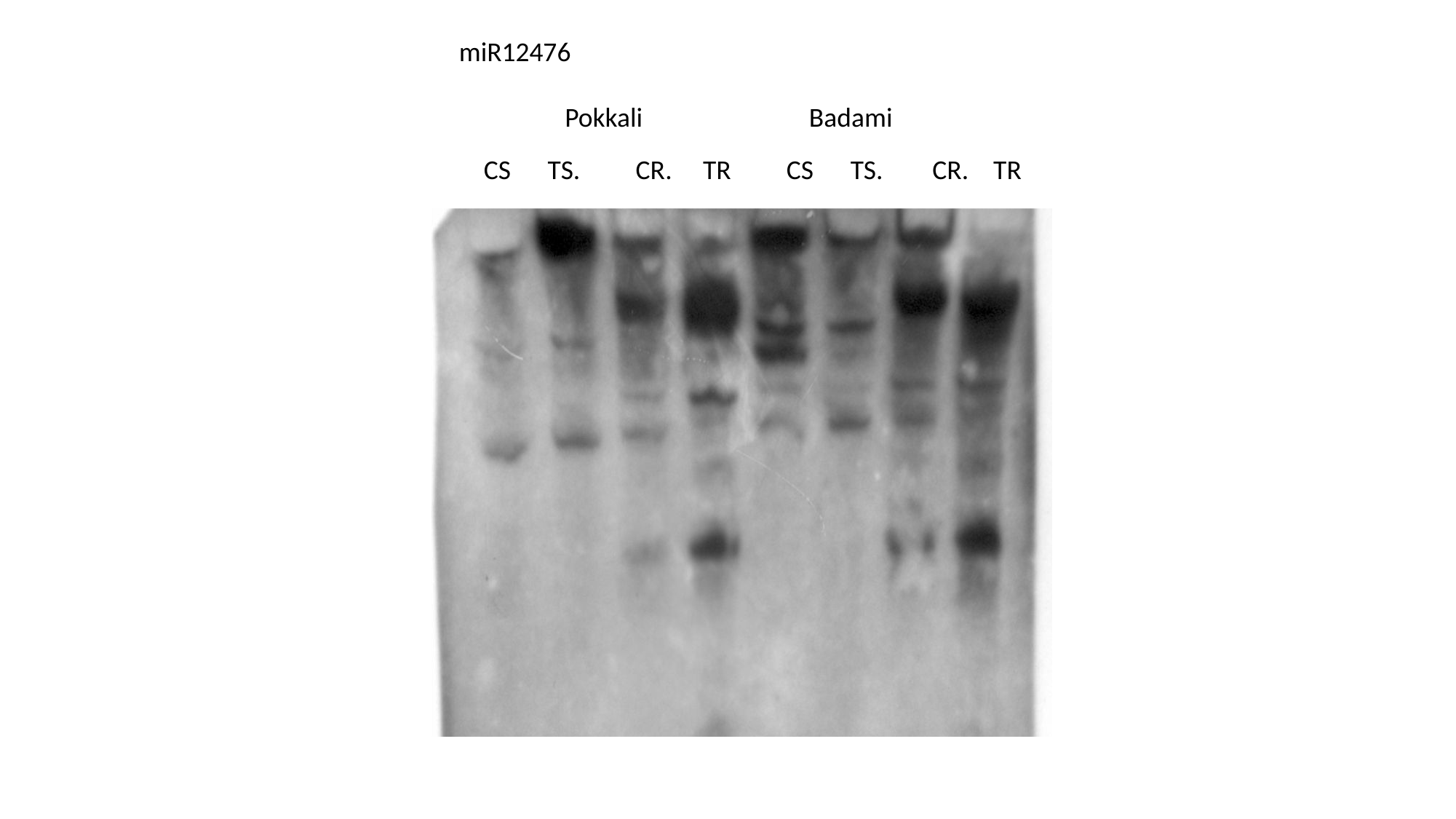

miR12476
Pokkali Badami
CS TS. CR. TR CS TS. CR. TR

## Slide 6
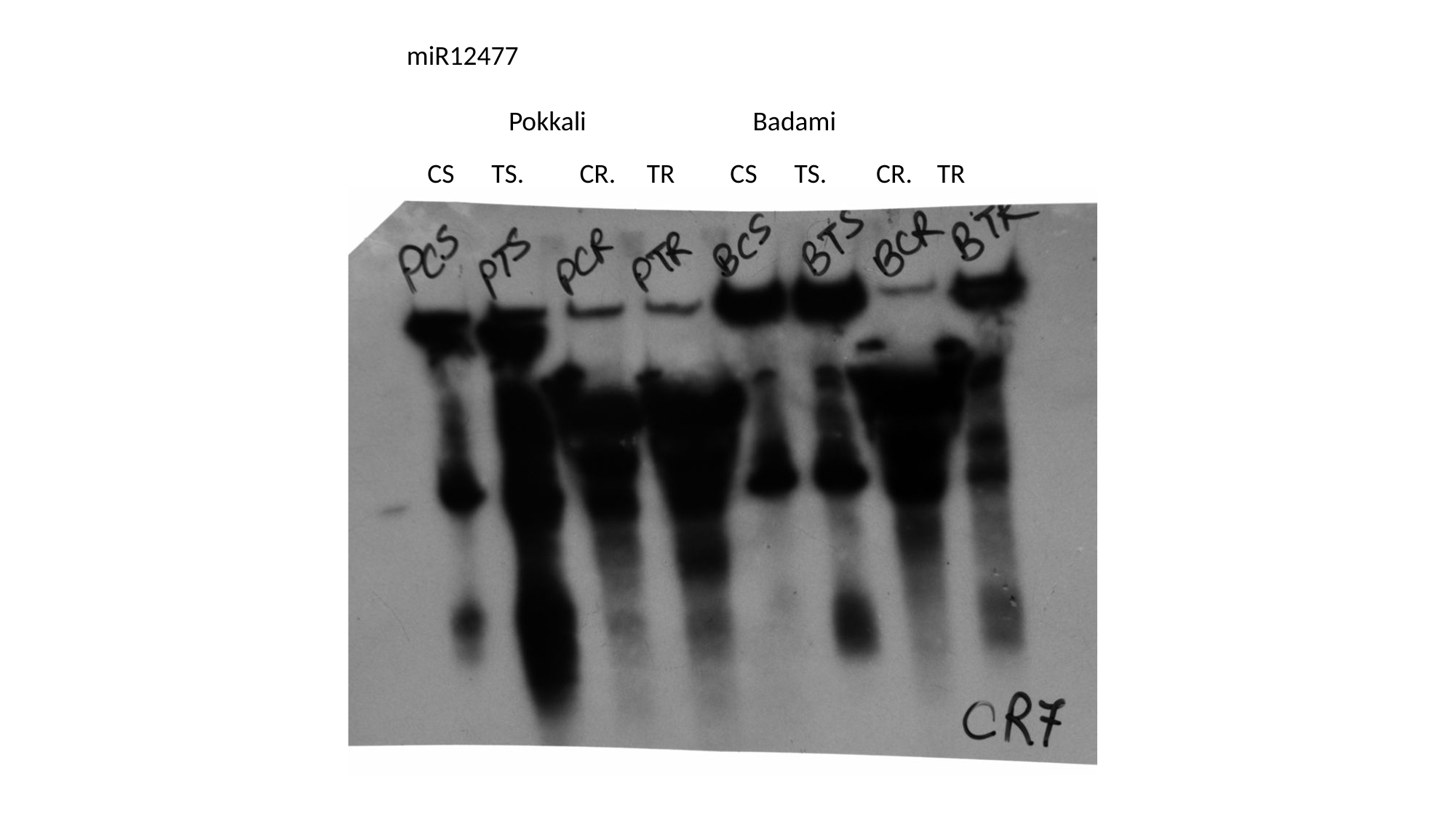

miR12477
Pokkali Badami
CS TS. CR. TR CS TS. CR. TR

## Slide 7
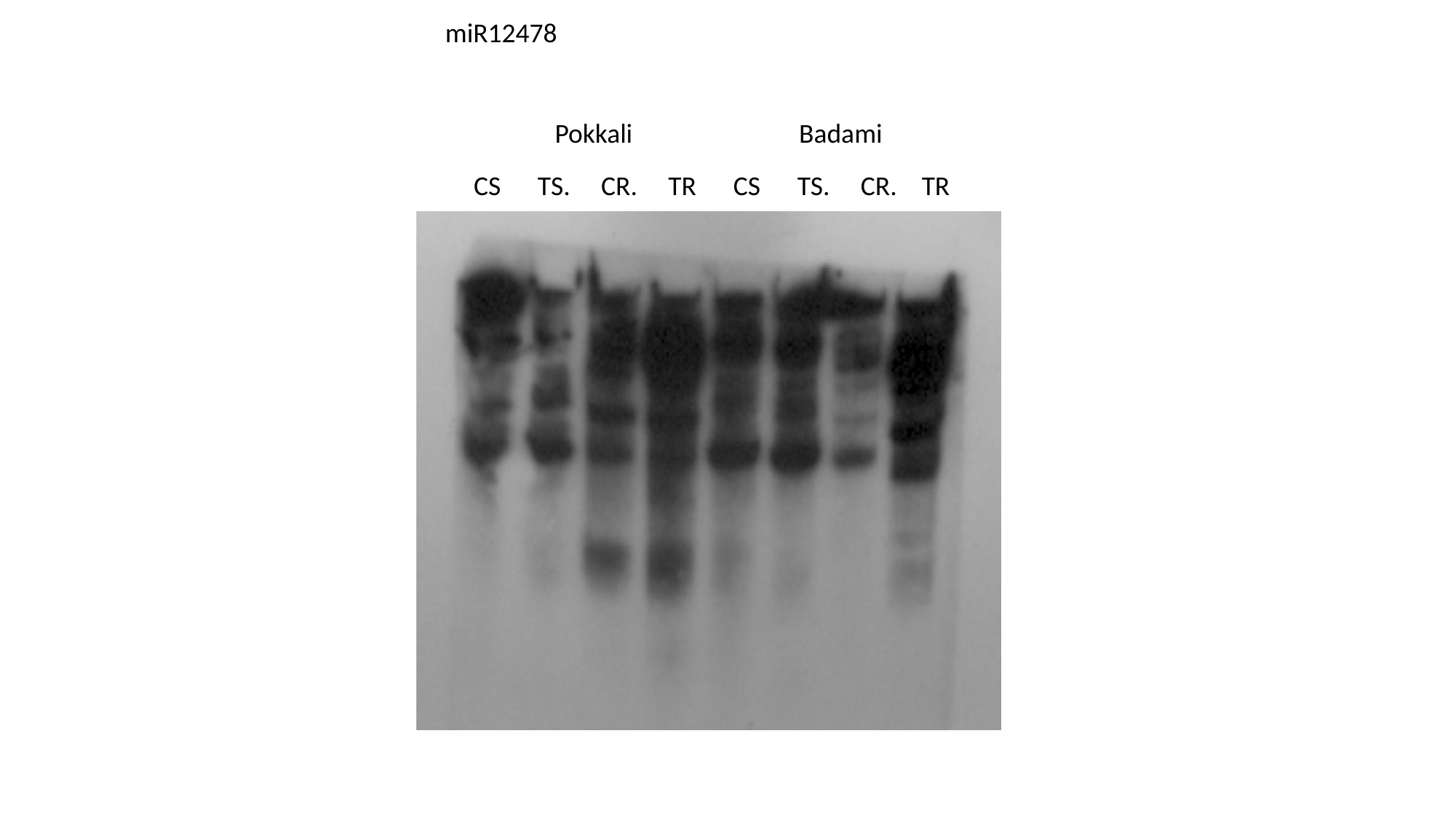

miR12478
Pokkali Badami
CS TS. CR. TR CS TS. CR. TR

## Slide 8
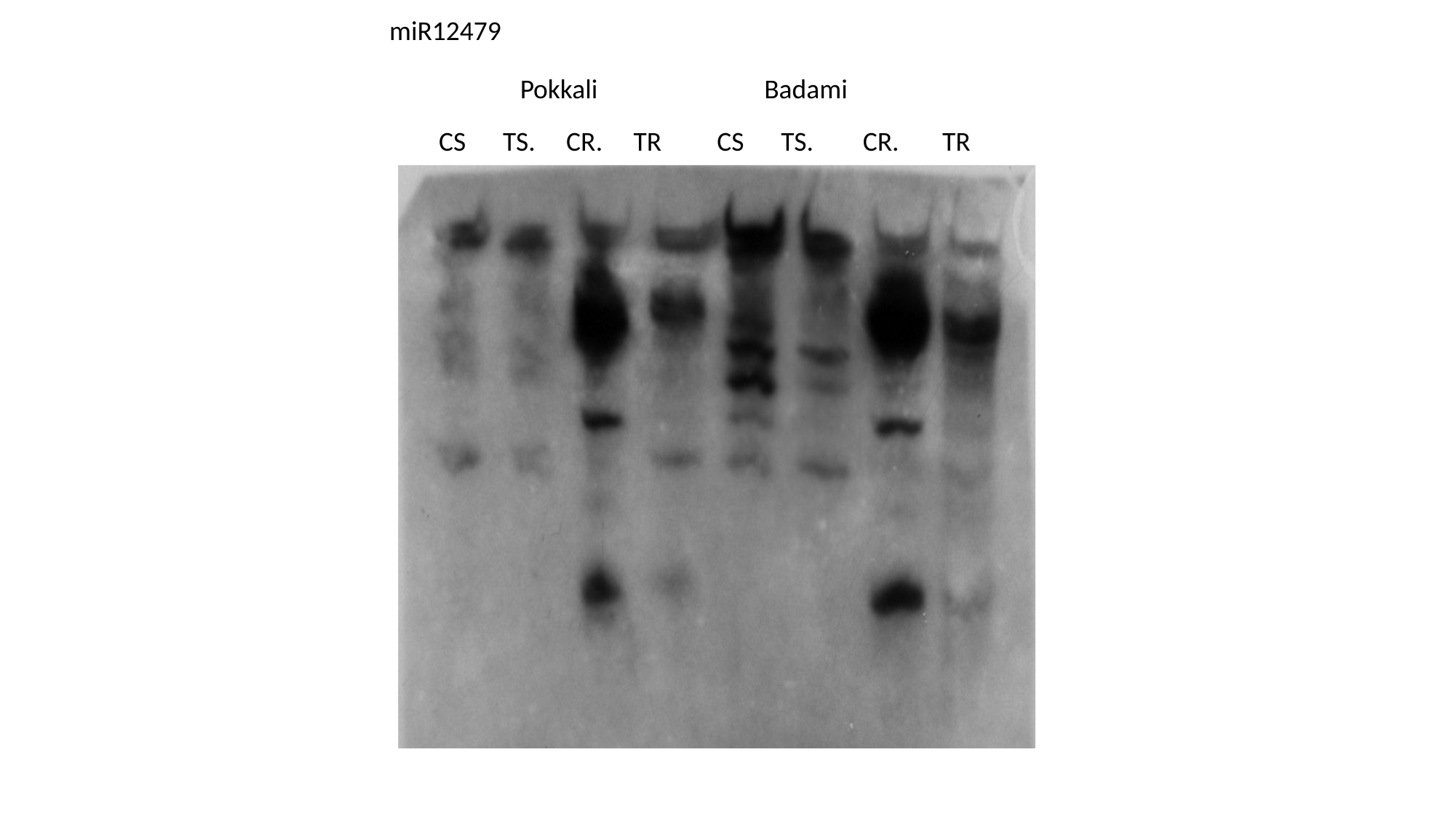

miR12479
Pokkali Badami
CS TS. CR. TR CS TS. CR. TR

## Slide 9
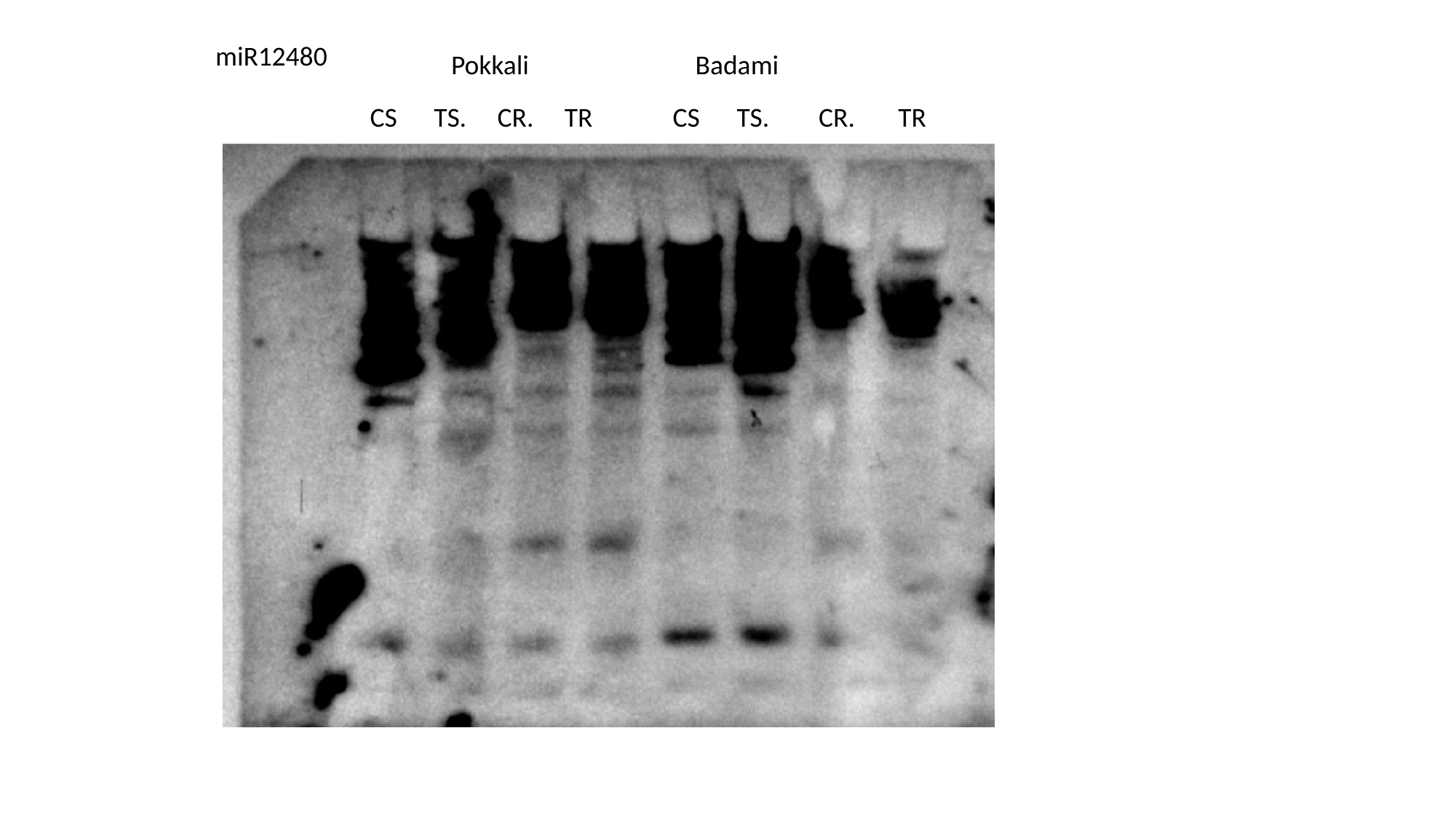

miR12480
Pokkali Badami
CS TS. CR. TR CS TS. CR. TR

## Slide 10
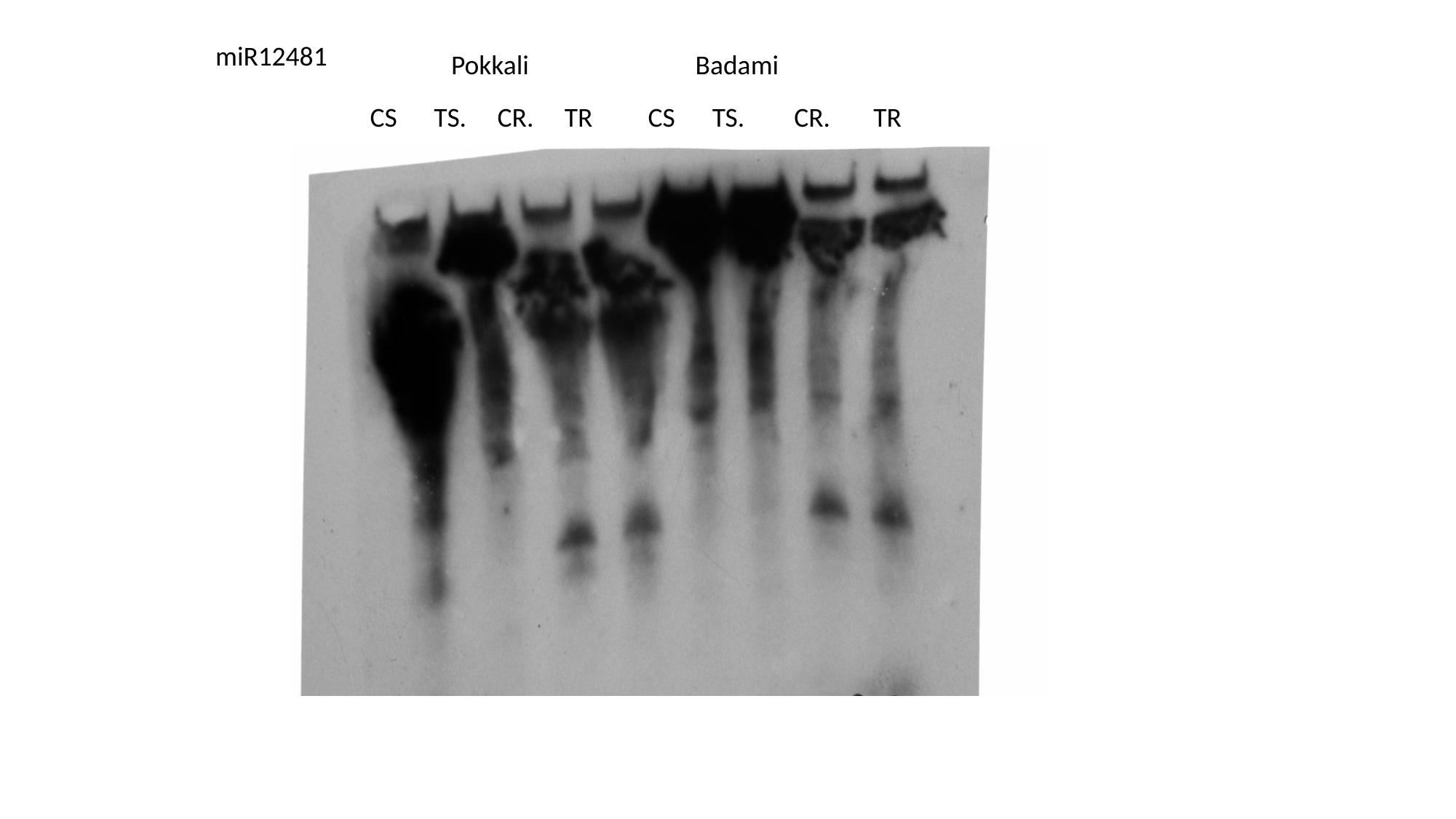

miR12481
Pokkali Badami
CS TS. CR. TR CS TS. CR. TR

## Slide 11
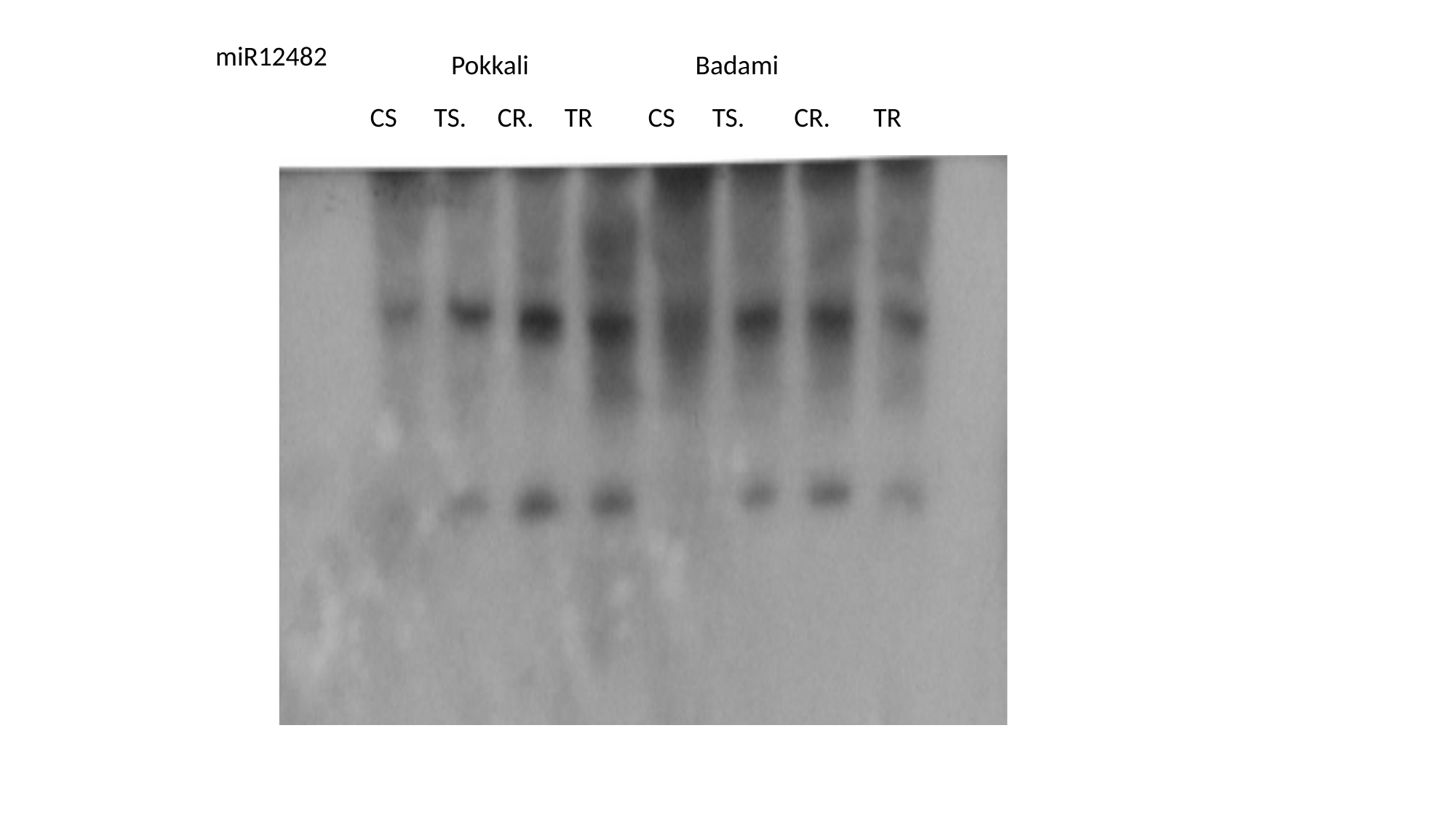

miR12482
Pokkali Badami
CS TS. CR. TR CS TS. CR. TR
