# Supplementary material for: Identification and expression analysis of miRNAs and elucidation of their role in salt tolerance in rice varieties susceptible and tolerant to salinity
Source: PLoS One. 2020 Apr 15;15(4):e0230958. doi: 10.1371/journal.pone.0230958 (PMC7159242; doi:10.1371/journal.pone.0230958)
Supplement: S6 File — (PPTX) [file pone.0230958.s006.pptx]

## Slide 1
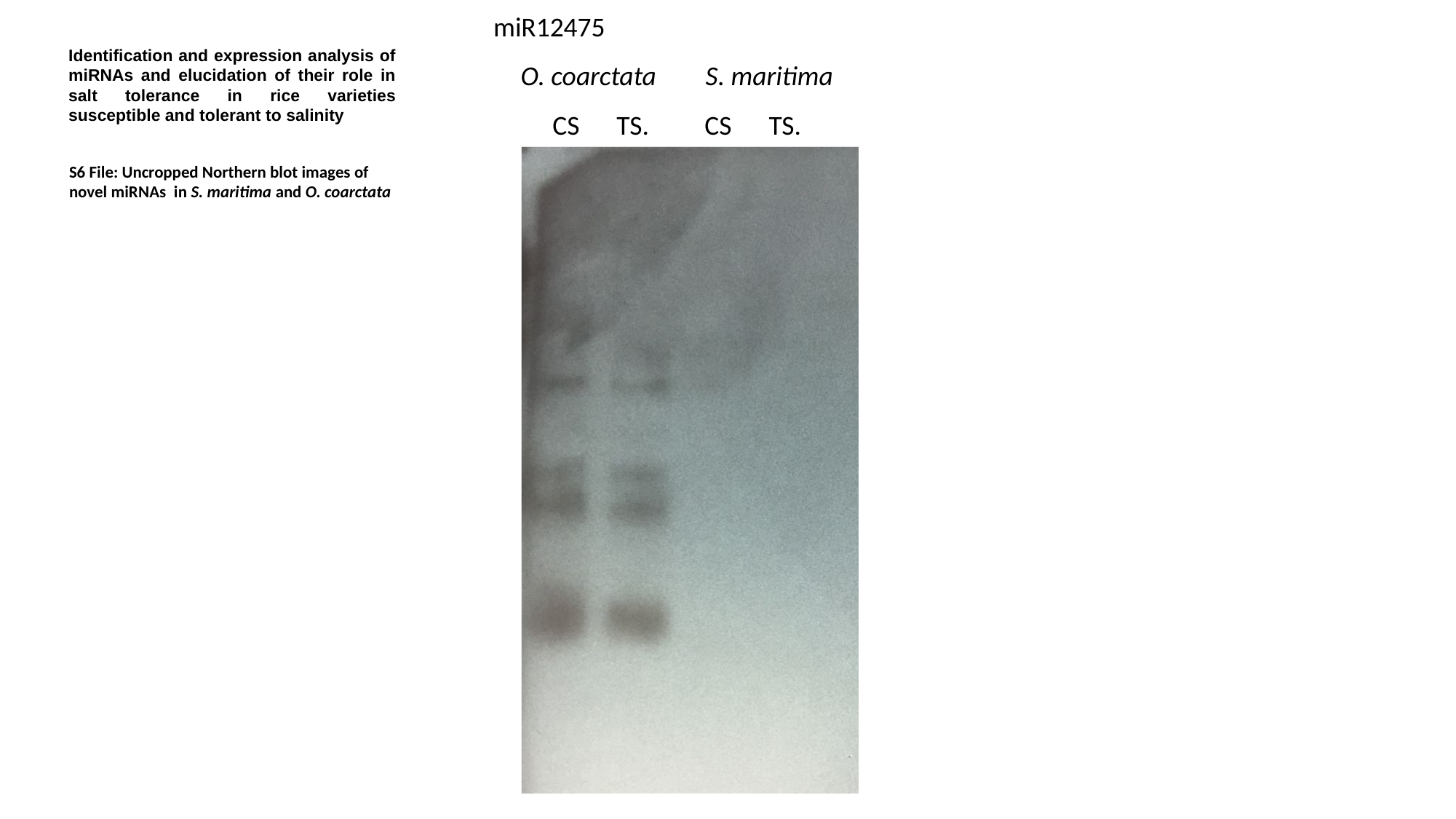

miR12475
CS TS. CS TS.
O. coarctata S. maritima
Identification and expression analysis of miRNAs and elucidation of their role in salt tolerance in rice varieties susceptible and tolerant to salinity
S6 File: Uncropped Northern blot images of
novel miRNAs in S. maritima and O. coarctata

## Slide 2
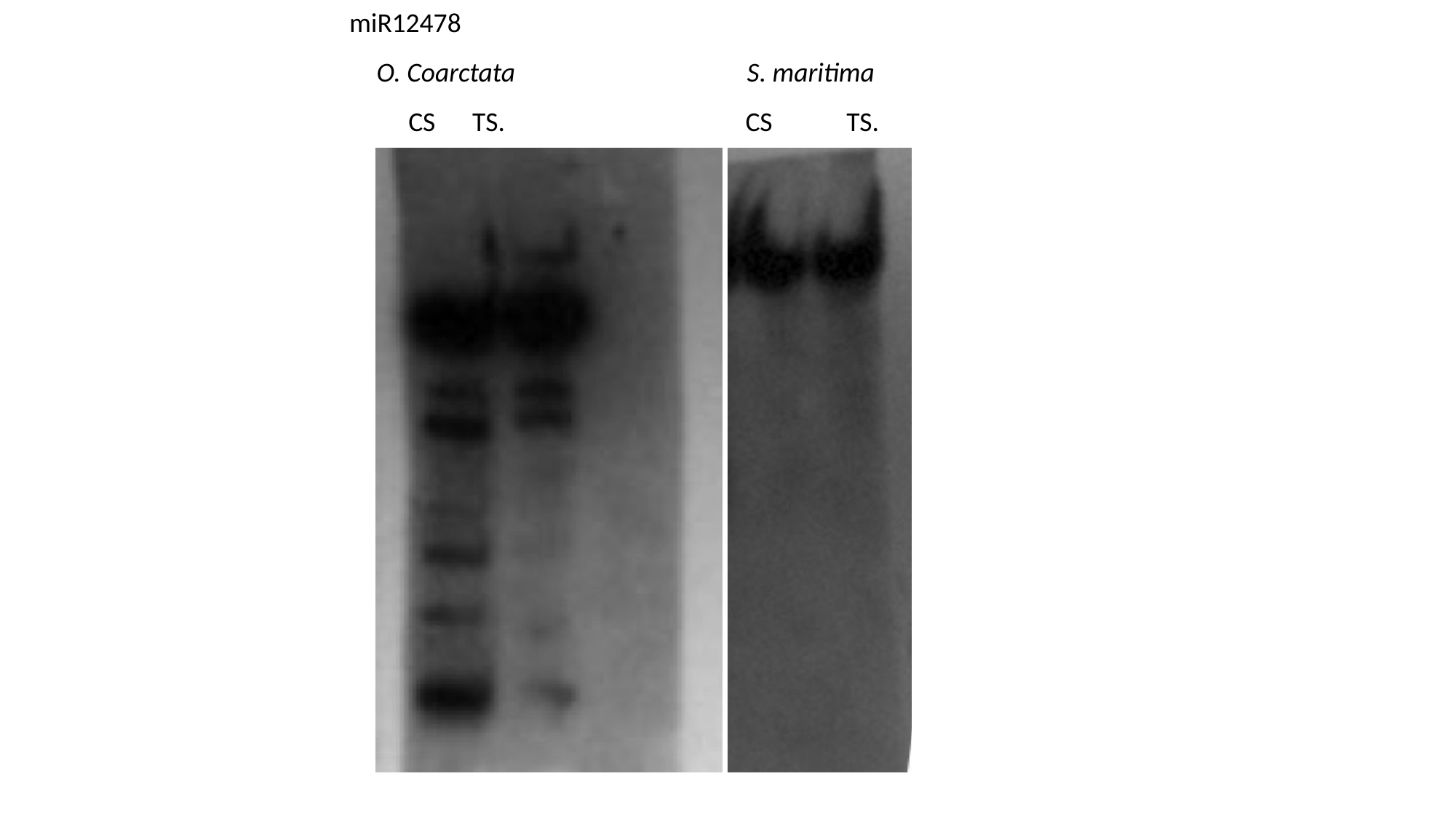

miR12478
CS TS. CS TS.
O. Coarctata 		 S. maritima

## Slide 3
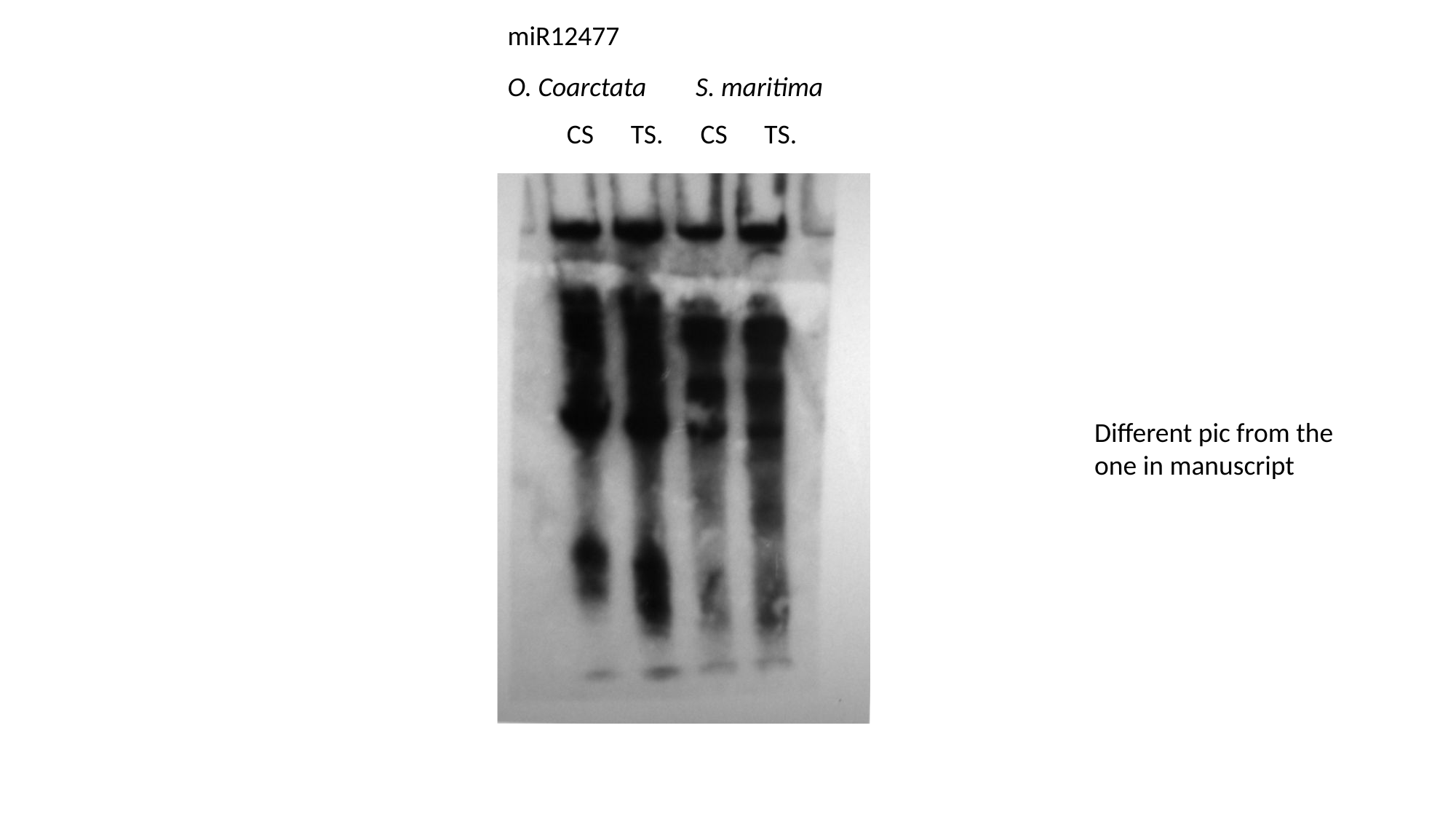

miR12477
CS TS. CS TS.
O. Coarctata S. maritima
Different pic from the one in manuscript

## Slide 4
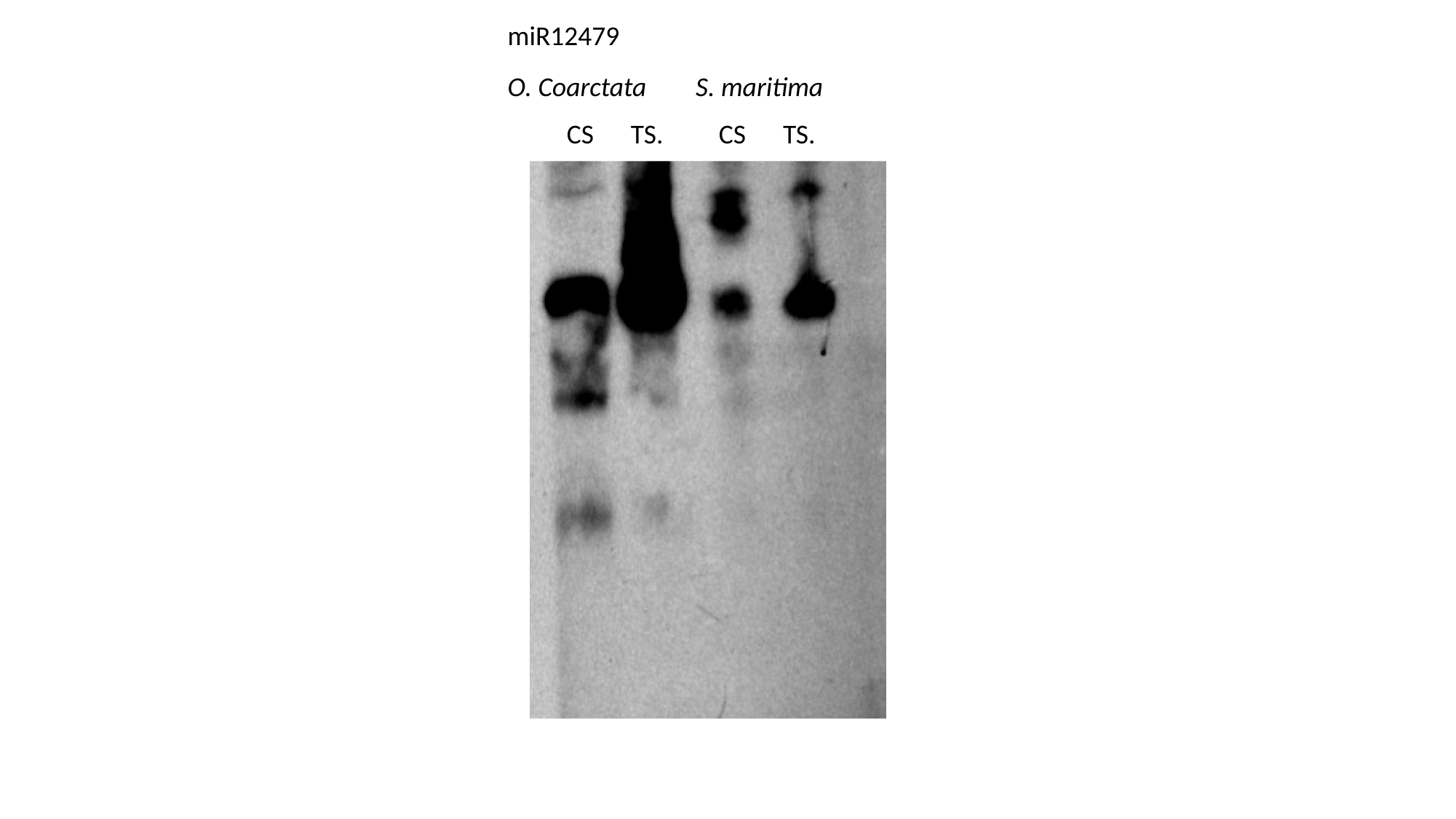

miR12479
CS TS. CS TS.
O. Coarctata S. maritima

## Slide 5
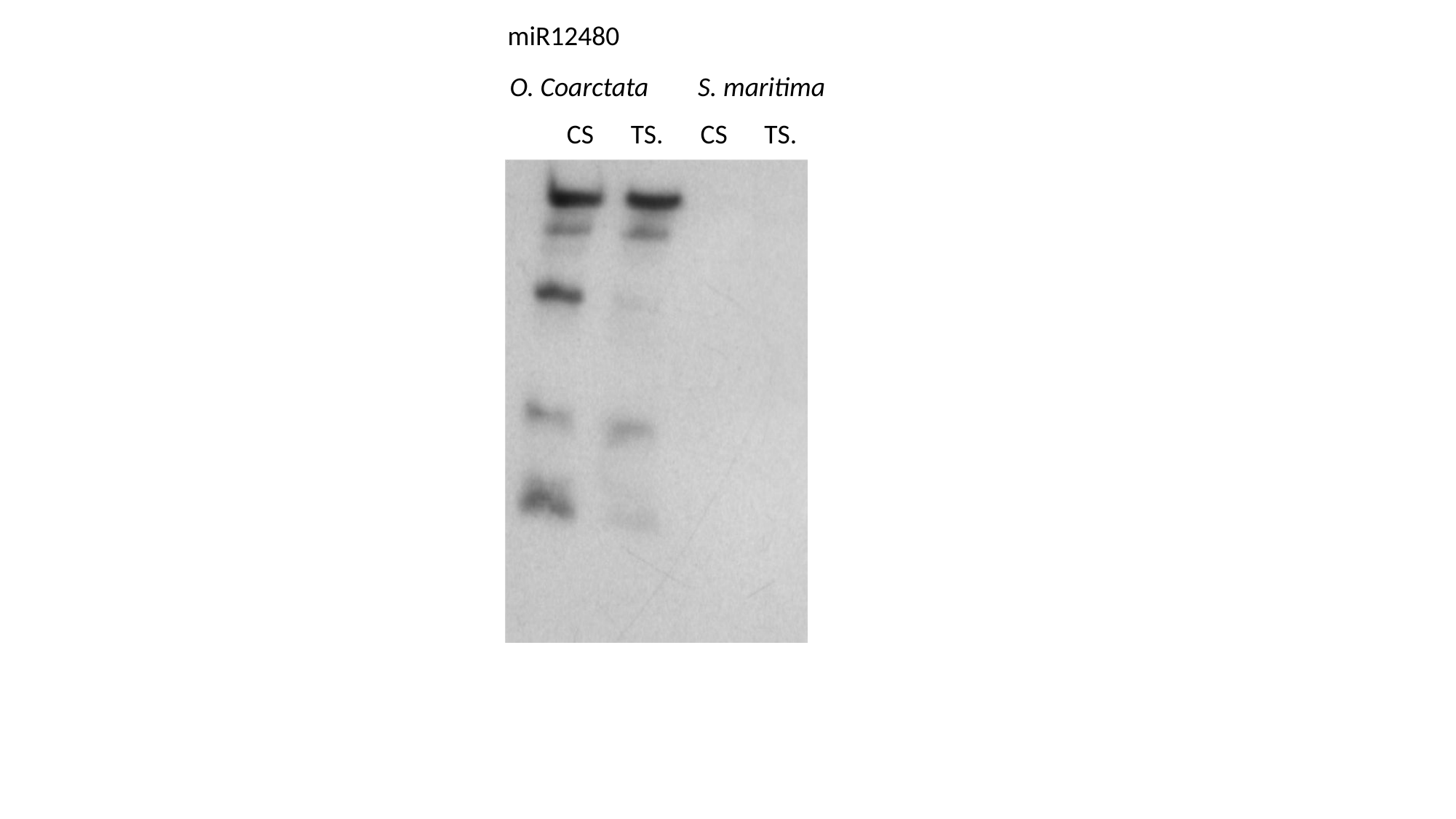

miR12480
CS TS. CS TS.
O. Coarctata S. maritima

## Slide 6
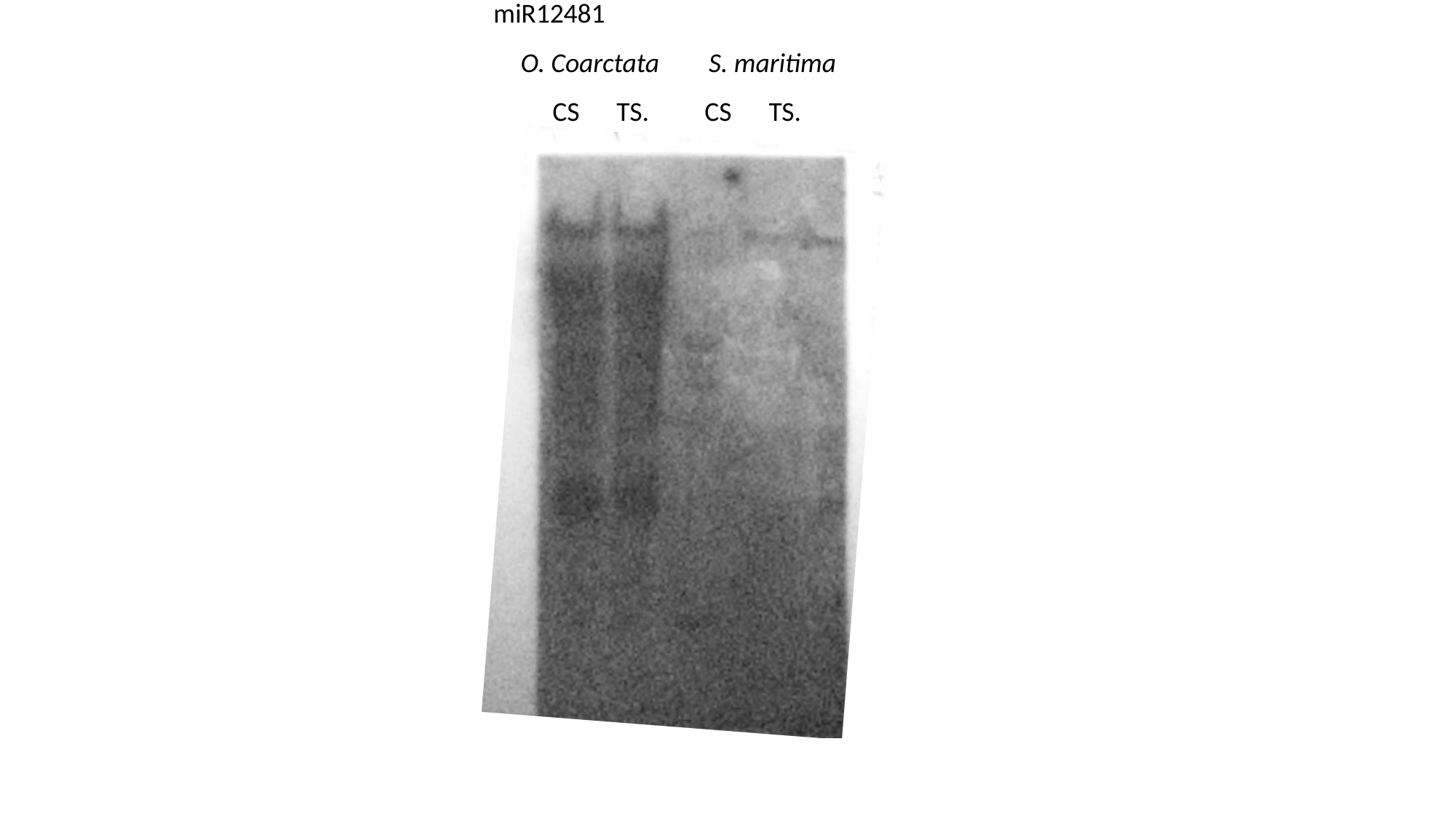

miR12481
CS TS. CS TS.
O. Coarctata S. maritima

## Slide 7
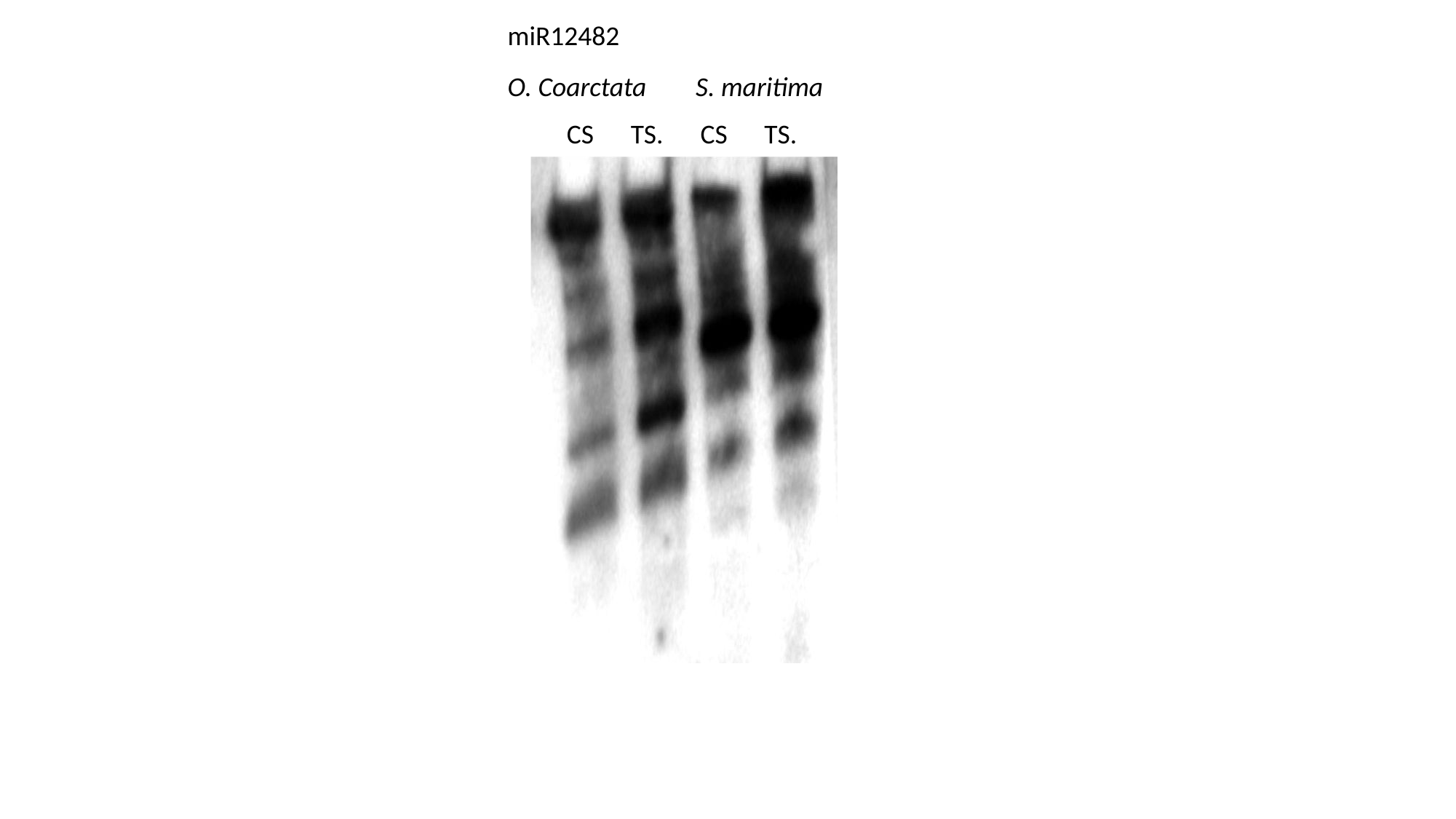

miR12482
CS TS. CS TS.
O. Coarctata S. maritima
